# Treatment of chronic anal fissure in Crohn’s disease patients with freshly collected autologous adipose tissue: a pilot study

**DOI:** 10.1007/s10151-025-03230-3

**Published:** 2025-11-23

**Authors:** Asmaa Sulaiman, Anders Dige, Andreas Hurup Nordholm, Lilli Lundby

**Affiliations:** 1 Department of Hepatology and Gastroenterology, Aarhus University Hospital, Aarhus, Denmark; 2 Digestive Disease Center, Bispebjerg Hospital, Copenhagen University, Copenhagen, Denmark; 3 Department of Surgery and Pelvic Floor Unit, Aarhus University Hospital, Palle Juul-Jensens, Boulevard 99, 8200 Aarhus N, Denmark

**Keywords:** Chron’s disease, Autologous adipose tissue, Anal fissure, Sphincter-preserving treatment

## Abstract

**Background:**

Chronic anal fissures in patients with Crohn’s disease (CD) remain a significant therapeutic challenge, particularly when linked to active perianal disease. Conventional treatments often fail, highlighting the need for alternative approaches. This study explores the efficacy and safety of freshly collected autologous adipose tissue injection (AATI) for treating chronic fissures in patients with CD.

**Methods:**

Nine patients with CD with anal fissures were included. The primary outcome was complete healing (CH) at 3 months after last AATI, defined as full fissure re-epithelialization and complete pain relief. Secondary outcomes included changes in defecation pain (visual analog scale [VAS]), anal discomfort (VAS), Perianal Disease Activity Index (PDAI), and St. Mark’s Incontinence Score (SMIS).

**Results:**

Five patients (56%) achieved CH after one (*n* = 4) or two (*n* = 1) AATI. Partial healing was observed in four patients (44%). Defecation pain improved from a VAS score of 7.5 (IQR 5.0–8.5) to 2.75 (0.0–4.5; *p* = 0.009), anal discomfort from VAS score of median 5.0 (2.5–6.5) to 1.0 (0.0–2.5; *p* = 0.014), PDAI from 5.0 (3.0–6.0) to 1.0 (1.0–2.0; *p* = 0.022), and SMIS from 7.0 (4.0–9.0) to 4.0 (0.0–4.0; *p* = 0.041). No treatment-related complications occurred.

**Conclusion:**

AATI may be a promising new treatment of chronic anal fissures in patients with CD. Effects of AATI should be explored further in controlled trials.

## Introduction

Anal fissures are longitudinal tears in the anoderm, often accompanied by pain and bleeding. Acute fissures heal within a few weeks, while chronic fissures are defined as those that persist for more than 8–12 weeks [1]. The pathogenesis is thought to involve local trauma and/or ischemia, which hinder the healing process.

While most anal fissures resolve with conservative, nonsurgical treatments such as stool-bulking agents and topical medications such as corticosteroids and diltiazem, which reduce sphincter tonus and inflammation, approximately 40% of fissures persist and become chronic [1, 2]. For refractory cases, surgical treatments such as botulinum toxin injections or lateral internal sphincterotomy (LIS) are often employed. Although LIS is more effective than botulinum toxin in promoting healing and preventing recurrence, the procedure carries a significant risk of both transient and permanent fecal incontinence [3].

In patients with Crohn’s disease (CD), chronic anal fissures may also arise owing to ongoing intestinal inflammation rather than isolated local trauma. Treating anal fissures in these patients is uniquely challenging, as conventional local therapies and botulinum toxin injections frequently fail to achieve healing [4]. Unhealed fissures in patients with CD can progress to complications such as perianal fistulas or abscesses, necessitating early surgical intervention. LIS is relatively contraindicated in patients with CD owing to the elevated risk of impaired wound healing and development of fecal incontinence [4, 5]. This highlights the necessity for novel and sphincter-preserving therapeutic modalities for chronic anal fissures in patients with CD.

A so far unexplored treatment of fissures in patients with CD is the use of freshly collected autologous adipose tissue injections (AATI). Two small studies have reported favorable outcomes from treating chronic anal fissures in patients without CD using either adipose-derived regenerative cells (ADRCs) or AATI [6, 7]. The use of ADRCs requires laboratory processing for cell isolation and can therefore not be performed as a single step procedure. Using freshly collected autologous adipose tissue may in this perspective serve as a more easily accessible treatment as it can be managed. We have previously demonstrated that freshly collected autologous adipose tissue injections may be effective in treating complex anal fistulas in Crohn’s disease. This experience encouraged us to explore the method in patients with chronic anal fissures [8, 9]. This pilot study aims to evaluate the efficacy and safety of treating chronic anal fissures in patients with CD with AATI.

## Methods and materials

### Study design and patients

This study is a prospective, observational study conducted at the Department of Surgery and Pelvic Floor Unit, Aarhus University Hospital and Digestive Disease Centre, Bispebjerg University Hospital, University of Copenhagen. A total of nine patients with CD with chronic anal fissures who received treatment with AATI were included.

The inclusion criteria required patients to be ≥ 18 years, have a confirmed diagnosis of CD, and present with one or more symptomatic chronic anal fissures (pain, bleeding, or soiling) persisting for > 8 weeks. All patients had previously received at least 4 weeks of conventional topical therapy with diltiazem and/or local corticosteroid without sufficient effect, and all topical treatments had been discontinued before inclusion. Two patients had additionally received botulinum toxin injection without benefit. None had undergone pelvic floor therapy prior to inclusion. Participants had received stable medical therapy for CD for at least 12 weeks prior to inclusion. Exclusion criteria were active rectal CD, malignant or chronic infectious disease, insulin-dependent diabetes, pregnancy, and smoking or smoking cessation within the previous 8 weeks.

### Surgical procedure

**Fig. 1:**
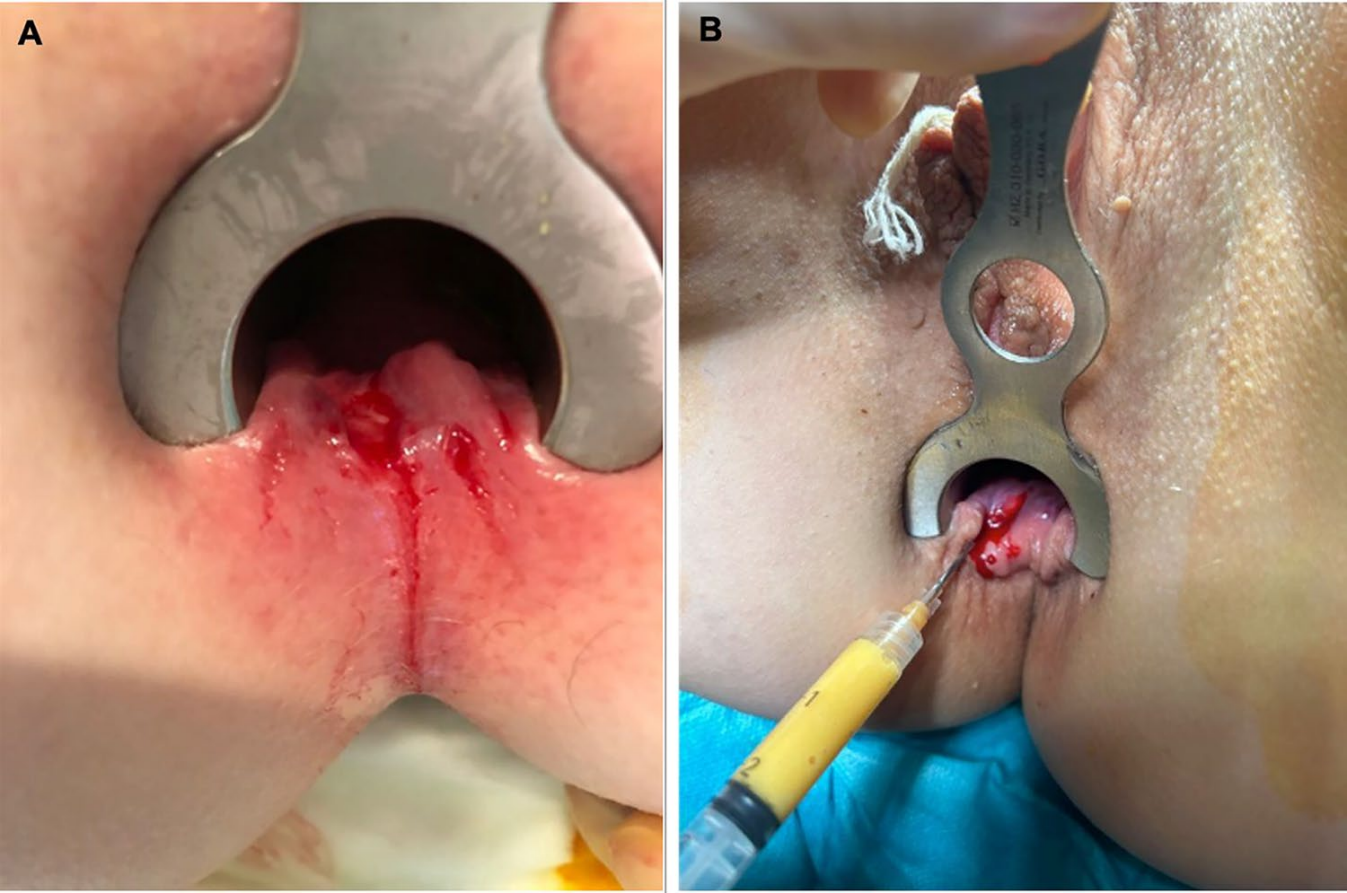
Overview of treatment procedure with autologous adipose tissue. **A** Clinical appearance of a chronic anal fissure in a patient with CD before autologous adipose tissue injection. The anoderm appears fragile with signs of superficial rhagades, consistent with chronic inflammation and tissue vulnerability. **B** Freshly collected adipose tissue injected into a CD fissure

Liposuction and injection of adipose tissue into the fissure were performed under general anesthesia by an experienced colorectal surgeon in one procedure. Prophylactic antibiotics (1.5 g cefalotin,1.5 g metronidazole, and 1 g of dicloxacillin) was given intravenously preoperatively according to local practice. A mixture of 1 mg of adrenaline was added to 1000 mL of Ringer’s solution. Then, 100–200 mL of this mixture was injected into the subcutaneous layer of the lower abdominal wall. Approximately 20 mL of adipose tissue was obtained from each patient. The harvested adipose tissue was centrifuged and subsequently manually microfragmented before being carefully injected into the fissure, as illustrated in Fig. 1. The process of harvesting and preparing the adipose tissue took approximately 25 min. All patients were prescribed oral painkillers on demand, ascorbic acid 1000 mg for 6 weeks, and laxatives according to the department’s regular postoperative regime following fissure surgery. Patients were discharged from the hospital a few hours after the procedure and followed up next day by telephone interview.

### Clinical evaluation and study endpoints

All patients underwent careful clinical inspection of the anal region prior to inclusion. At the time of the procedure, patients were examined under general anesthesia to re-evaluate the fissure and to exclude the presence of an underlying fistula or abscess. In cases of diagnostic uncertainty, perioperative endoanal ultrasound or preoperative pelvic magnetic resonance imaging (MRI) was performed to support the clinical assessment. Anorectal manometry was not performed, as hypertonia of the internal anal sphincter is not considered a main pathogenic factor in CD-associated fissures and the method is technically challenging and often unreliable in this painful patient group.

A fissure was defined as a longitudinal, sharply demarcated lesion starting below the dentate line, often with exposure of internal sphincter fibers. Healing was defined as complete re-epithelialization of the fissure at anal inspection combined with complete pain relief. Fissure healing was assessed by visual inspection at follow-up visits.

Follow-up visits were scheduled at 1, 3, 6, and 12 months after treatment. The primary outcome was complete healing (CH) of the fissure 3 months after the last injection. Patients without CH at 3 months after the first treatment were offered a second injection of freshly collected autologous adipose tissue or, alternatively, another treatment modality. Patients achieving CH 3 months after the second injection were likewise considered to have met the primary outcome. Partial healing (PH) was defined as clinical improvement, characterized by either reduced fissure size or decreased pain, without meeting the criteria for CH.

The secondary outcomes were (1) reduction in defecation pain, measured by visual analog scale (VAS) at baseline and at 1, 3, 6, and 12 months; (2) reduction in overall anal discomfort, also assessed by VAS at the same time points; (3) improvement in perianal CD activity, measured by the Perianal Disease Activity Index (PDAI) at baseline and follow-up visits; and (4) changes in fecal continence function, evaluated using the St. Mark’s Incontinence Score (SMIS) at baseline and follow-up.

### Statistical analyses

Continuous variables are summarized using the median and interquartile range (IQRs) owing to the nonnormal distribution of the data. Categorical outcomes are presented as absolute numbers and percentages. Changes in clinical symptom scores between baseline and 3 month follow-up were analyzed using the Wilcoxon signed-rank test, a nonparametric method for paired data. This included comparisons of defecation pain, anal discomfort, PDAI, and SMIS. *p*-Value < 0.05 was considered statistically significant. All statistical analyses were performed using R (version 5.4.1; R Core Team, 2025).

### Ethical considerations

The study was approved by the local Ethical Committee (journal number 1-10-72-151-22).

All patients provided written informed consent prior to participation.

## Results

### Patient characteristics

Nine patients were enrolled in the study, consisting of eight women and one man. The median age of the cohort was 28 years (IQR: 26–53 years). The fissures had been present for a median duration of 14 months (IQR: 12–208 months) prior to first AATI. At the time of treatment, five patients received treatment with anti-tumor necrosis factor-α (TNF-α) antibodies (infliximab [*n* = 3]; adalimumab [*n* = 2]). Two patients were also receiving concomitant immunosuppressive therapy with azathioprine. One patient received monotherapy with vedolizumab, and one with risankizumab. Two patients were not receiving any systemic treatment. Eight patients received a single AATI, while one patient received two AATIs. The median volume of injected adipose tissue was 7 mL (IQR: 5.0–15 mL) per treatment. Patient characteristics are presented in Table 1.

**Table 1:** Baseline characteristics and result of injections with freshly collected autologous adipose tissue

Patient number	Sex	Age	Medical treatment	Duration of fissure (months)	AATI (*n*)	Volume of injected adipose tissue (ml)	Results
1	Male	28	Adalimumab	21	1	7,5	PH
2	Female	30	Infliximab	12	1	5	CH
3	Female	25	Infliximab azathioprine	24	1	3	PH
4	Female	21	Risankizumab	50	2	First: 2 Second: 8	CH
5	Female	53	None	208	1	15	CH
6	Female	26	Infliximab azathioprine	14	1	5	PH
7	Female	27	None	14	1	9	PH
8	Female	34	Adalimumab	6	1	6	CH
9	Female	38	Vedolizumab	7	1	10	CH

*AATI* autologous adipose tissue injection, *PH* partial healing, *CH* complete healing

### Effects of treatment with autologous adipose tissue injections

#### Primary outcome

At 3 months after a single AATI, four (44%) of the nine total patients had achieved CH. One additional patient had achieved CH 3 months after a second injection, resulting in a total of 56% of patients who achieved the primary outcome.

Among patients who did not achieve CH, four (44%) experienced partial healing. These patients reported from 40% to 75% reduction in pain at 3 months, accompanied by clinically reduced fissure size. One of these patients had achieved complete pain relief after 3 months, but only partial epithelization was observed by clinical examination. This patient reported further improvement and demonstrated complete epithelization at the 12 month follow-up.

#### Secondary outcomes

Clinical improvement was observed across all secondary outcomes. Defecation pain, anal discomfort, PDAI score, and SMIS all showed reductions following treatment, with the most pronounced improvements seen within the first 3 months.

At 3 months, the median defecation pain score decreased from 7.5 (IQR 5.0–8.5) at baseline to 2.75 (IQR 0.0–4.5), and anal discomfort decreased from 5.0 (IQR 2.5–6.5) to 1.0 (IQR 0.0–2.5). The PDAI score was reduced from a median of 5.0 (IQR 3.0–6.0) to 1.0 (IQR 1.0–2.0), while SMIS improved from 7.0 (IQR 4.0–9.0) to 4.0 (IQR 0.0–4.0). All changes were statistically significant (*p* = 0.009 for defecation pain, *p* = 0.014 for anal discomfort, *p* = 0.022 for PDAI, and *p* = 0.041 for SMIS) (Table 2).

**Table 2:** Changes in secondary outcomes over time

Time	Defecation pain (VAS score)	Anal discomfort (VAS score)	PDAI-score	SMIS
Baseline	7.5 (5.0–8.5)	5.0 (2.5–6.5)	5.0 (3.0–6.0)	7.0 (4.0–9.0)
1 month (*n* = 5)	2.0 (1.0–4.0)	1.0 (1.0–2.5)	3.0 (1.0–3.0)	4.0 (4.0–7.0)
3 months (*n* = 9)	2.75 (0.0–4.5)	1.0 (0.0–2.5)	1.0 (1.0–2.0)	4.0 (0.0–4.0)
6 months (*n* = 4)	3.25 (2.5–4.0)	1.5 (1.125–2.375)	3.0 (2–0-5.75)	2.0 (1.5–3.0)
12 months (*n* = 4)	0 (0–0)	1.25 (0.75–1.875)	1.0 (0.0–3.0)	0.0 (0.0–1.0)

Values presented as median (IQR: 25th–75th percentile)

*n* number of patients assessed at each time point

Defecation pain improved after baseline in all patients (100%) at each follow-up point, while anal discomfort had improved in 80% of the patients at 1 month, increasing to 100% at 3 and 6 months, and 75% at 12 months. PDAI scores decreased in all patients at 1 and 3 months but showed less consistent improvements at later time points (25% at 6 months, 50% at 12 months). SMIS had improved in 80% of patients at 1 month, 89% at 3 months, and 100% at both 6 and 12 months (Fig. 1).

**Fig. 2:**
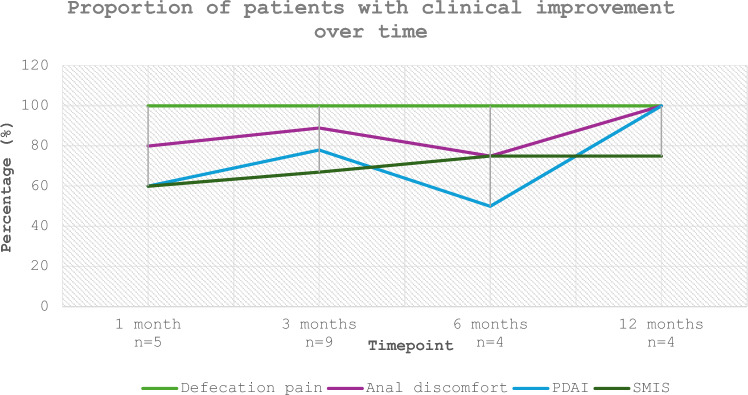
Proportion of patients demonstrating clinical improvement over time across secondary outcomes. Clinical improvement was defined as a reduction in score compared with baseline. The graph illustrates the percentage of patients with improvement at 1, 3, 6, and 12 months for each of the following parameters: defecation pain (VAS), anal discomfort (VAS), PDAI, and SMIS

Together, these findings support a substantial early treatment effect across multiple clinical domains, with maintained or partially sustained improvements at longer-term follow-up in most patients (Fig. 2).

### Complications and safety

No adverse events or treatment-related complications were observed during the study period. However, two patients experienced worsening in luminal CD during the follow-up period. This was not considered related to the treatment with AATI. One of these patients developed an intersphincteric fistula despite healed fissures. No incontinence was observed as a complication related to the AATI. However, one patient showed a worsened SMIS, which was attributed to pharmacological treatment for constipation and did not reflect a true deterioration in continence.

## Discussion

To our knowledge, this is the first study to evaluate the use of AATIs as a therapeutic approach for chronic anal fissures in patients with CD. The procedure was well tolerated, and no treatment-related complications were observed, supporting a favorable preliminary safety profile.

Complete healing was achieved in five of nine patients (56%) within 3 months after treatment, although one patient required two injections to achieve this outcome. Among the remaining four patients, partial healing was observed with reductions in fissure size and substantial improvements in pain. Importantly, nearly all patients reported symptomatic benefit, including reduced anal discomfort, improved PDAI scores, and better continence function (SMIS). These clinical improvements suggest that AATI may offer symptom relief even in the absence of complete epithelialization.

While the healing rate may appear modest, it should be interpreted in the context of CD-associated fissures, which are notoriously difficult to manage, often refractory to conventional topical therapies, and prone to relapse. According to the study protocol, all patients had already experienced no healing and only limited effect from at least 4 weeks of standard topical medical treatment prior to inclusion, underscoring that this was a treatment-refractory cohort. Notably, two patients had also failed botulinum toxin injections prior to enrollment. In this challenging setting, achieving complete healing in about half of the patients and clinically meaningful symptom improvement in the majority may be regarded as an encouraging result, supporting the feasibility of further exploration.

Several studies have explored the use of mesenchymal stromal cells and adipose-derived therapies for treating perianal disease in CD, particularly complex fistulas, with promising results [8]. However, data on the use of stem cells or autologous adipose tissue for the treatment of chronic anal fissures in patients with CD remain scarce. The present study indicates that even freshly collected adipose tissue—without laboratory processing—may induce clinical benefit in the majority of patients.

The observed improvements in secondary outcomes, including reductions in anal discomfort, PDAI, and SMIS, are consistent with the proposed anti-inflammatory and regenerative properties of adipose tissue. These effects may relate to both mechanical and biological mechanisms: the injected fat could provide a physical bulking effect that supports tissue healing, while bioactive components within the adipose tissue may modulate local immune responses and promote regeneration. The exact contribution of each mechanism remains uncertain, and the relative importance of structural versus biological effects cannot be determined from this pilot study [10, 11].

This study has several strengths, including prospective data collection, standardized clinical follow-up, and the use of clearly defined endpoints relevant to both patient-reported outcomes and objective healing. Nevertheless, important limitations must be acknowledged. This study is limited by its small sample size and lack of a control group, which preclude definitive conclusions about efficacy. The open-label design may introduce observational bias, and natural fluctuations in disease activity among patients with CD could have influenced some outcomes. This is particularly relevant in the context of anal fissures in CD, which—similar to luminal disease—tend to wax and wane over time, making permanent healing inherently more difficult compared with fistulizing disease, where more durable closure is often observed. The heterogeneity in CD maintenance therapies may also have influenced fissure healing, but this could not be assessed in this pilot study.

Furthermore, the small sample size restricts the statistical power. The statistical analyses applied in this study should therefore be regarded as exploratory and hypothesis-generating rather than definitive. We deliberately used medians, interquartile ranges, and the Wilcoxon signed-rank test, as these nonparametric methods are appropriate for small, non-normally distributed datasets and provide a more objective evaluation of trends than descriptive reporting alone. The purpose of including a statistical evaluation was not to demonstrate efficacy but to provide preliminary signals of feasibility and to inform the design and sample size calculation of future controlled trials.

Another limitation is that we did not perform a systematic assessment of central or peripheral sensitization (e.g., using the Central Sensitization Inventory) or collect information on comorbid chronic pain syndromes. As these factors may influence symptom perception and interfere with recovery in chronic anorectal conditions, this omission could have impacted our findings.

Finally, while all nine patients had follow-up data at 3 months—the primary outcome time point for assessing complete healing and pain relief—extended follow-up beyond this point was only available for five patients. This limited long-term follow-up reduces the ability to assess the durability of treatment response and may bias the results toward a more favorable interpretation, as late relapses or a fading treatment response might not be captured.

In conclusion, this pilot study suggests that freshly collected autologous adipose tissue injection may be a feasible and well-tolerated treatment option for chronic anal fissures in CD unresponsive to standard topical therapies. Approximately half of the patients achieved complete healing, and the majority reported symptomatic improvement. These preliminary findings should be interpreted with caution given the methodological limitations, and larger controlled studies are needed to confirm efficacy, define the role of AATI relative to conventional therapies, and optimize patient selection.

## Data Availability

The datasets generated and analyzed during the current study are not publicly available owing to ethical restrictions but are available from the corresponding author on reasonable request. Data were prospectively collected and managed using REDCap electronic data capture tools hosted at Aarhus University Hospital.
